# Diet of Critically Endangered Black‐Eyed Bent‐Toed Gecko, *Cyrtodactylus nigriocularis*, Nguyen, Orlov & Darevsky, 2006 From Vietnam

**DOI:** 10.1002/ece3.71645

**Published:** 2025-07-04

**Authors:** Hanh Thi Ngo, Minh Duc Le, Trang Thu Hoang, Ha Hoang Nguyen, Huy Quoc Nguyen, Quyen Hanh Do, Hanh Minh Vu Nguyen, Truong Quang Nguyen, Thomas Ziegler, Minh Le, Anh Van Pham

**Affiliations:** ^1^ Central Institute for Natural Resources and Environmental Studies Vietnam National University Hanoi Vietnam; ^2^ Institute of Zoology University of Cologne Cologne Germany; ^3^ AG Zoologischer Garten Köln Köln Germany; ^4^ Faculty of Environmental Sciences University of Science, Vietnam National University, Hanoi Hanoi Vietnam; ^5^ Institute of Biology Vietnam Academy of Science and Technology Hanoi Vietnam; ^6^ Mountain Ecological Restoration and Biodiversity Conservation Key Laboratory of Sichuan Province, Chengdu Institute of Biology, Chinese Academy of Sciences Chengdu China; ^7^ University of Chinese Academy of Sciences Beijing China; ^8^ HUS High School for Gifted Students Hanoi Vietnam; ^9^ Graduate University of Science and Technology Vietnam Academy of Science and Technology Hanoi Vietnam; ^10^ Department of Herpetology American Museum of Natural History New York New York USA

**Keywords:** Ba Den Mountain, invertebrates, prey items, stomach contents, Tay Ninh Province

## Abstract

The Black‐eyed Bent‐toed Gecko, *Cyrtodactylus nigriocularis* , a species endemic to Ba Den Mountain Cultural and Historical Complex, Tay Ninh Province, Vietnam, has been classified as Critically Endangered in the IUCN Red List since 2018. However, knowledge of its natural history is virtually non‐existent. To fill this gap, the diet of the gecko was studied by stomach flushing. We identified a total of 22 prey categories with 407 items in the stomachs of *C. nigriocularis* . The most important (IRI) groups among its prey were Araneae (24.33%), followed by Opiliones (16.59%), Achatinidae (10.67%), Blattidae (8.77%), Scolopendridae (7.59%), and Acrididae (4.20%), similar to food items consumed by tropical geckos as reported in previous studies. There was no relationship between body mass and mouth width of the species and length/volume of prey consumed, but there were significant differences in the diet composition between sexes and between age groups. Despite the discrepancies, spiders are important prey of all groups. In addition to furthering our knowledge of this poorly studied lizard, the research results can help design *ex situ* conservation measures for the species in case the wild population continues to decline as a result of local anthropogenic threats.

## Graphical abstract

Tropic niche of the Critically Endangered Black‐eyed Bent‐toed Gecko.

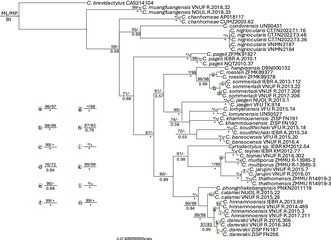

## 1 Introduction

Diet plays an important role in the daily life of animals, as it serves as an essential source of energy for growth, maintenance, and reproduction (Huey and Pianka 1981; Dunham et al. 1989; Zug et al. 2001). Animals usually specialize in different prey items and develop complex feeding behaviors based on dietary requirements or their anatomy (Schwenk 2002). Studying food composition can thus provide crucial information on ecological roles and the relative importance of each prey item in their diet (Losos and Greene 1988; Ortega‐Rubio et al. 1995; Znari and El Mouden 1997; Norval et al. 2012; Tan et al. 2020). In addition, dietary analysis in the context of interspecific and intraspecific interactions may shed light on niche overlap and how species partition their resources (Ortega‐Rubio et al. 1995; Rocha and Anjos 2007; Viieira and Port 2006; Bulté et al. 2008; Norval et al. 2012). Moreover, understanding their diet could help inform *ex situ* conservation efforts by determining the nutritional needs in captivity and develop more effective conservation measures for threatened species (Antwis et al. 2014; Chatpongcharoen et al. 2021; Gao et al. 2023).

The genus *Cyrtodactylus* Gray, 1827 is the most diverse radiation of the family Gekkonidae with more than 380 recognized species (Uetz et al. 2025). This widely distributed group occurs from West India through Bangladesh, southern China southwards to Malaysia, Indonesia, Sundas, Bali, the Philippines, and the Solomon Islands (Wood et al. 2012; Grismer et al. 2021, 2022; Uetz et al. 2025). Vietnam has long been recognized as a hotspot for new bent‐toed gecko discoveries (Grismer et al. 2021; Ngo et al. 2022). Since 1997, 52 new species of *Cyrtodactylus* have been described, making it a total of 55 known species (Uetz et al. 2025).

However, most of its members have been only found at their type localities within their specialized microhabitats or isolated islands. For example, *C. takouensis* is restricted to a few small granite caves in Ta Kou Nature Reserve; *C. phuquocensis* is only recorded in Phu Quoc Island; and *C. culaochamensis* only occurs in Cu Lao Cham Island (Ngo and Bauer 2008; Ngo et al. 2010; Ngo et al. 2020; Uetz et al. 2025). Consequently, many of them are exceptionally vulnerable to anthropogenic threats, such as habitat loss and degradation. Several species in Vietnam are facing exceedingly high extinction risks, including three taxa categorized as Critically Endangered, three as Endangered, and six as Vulnerable in the IUCN Red List (2025). To date, data on their ecology and population biology are scant. Until now, no study has been conducted to investigate the diet of the threatened species with a view to better understanding their trophic niche and designing appropriate *ex situ* conservation measures.

*C. nigriocularis* was described in 2006 based on the type series collected in Ma Thien Lanh Valley of Ba Den Mountain Cultural and Historical Complex (MCHC), Tay Ninh Province, southern Vietnam (Nguyen et al. 2006; IUCN Red List 2025). This species has been listed as Critically Endangered (CR) in the IUCN Red List since 2018 (Nguyen et al. 2018; Nguyen et al. 2019). Previous studies at its type locality in Tay Ninh Province only detected a handful of individuals, including ten during the first survey in 2005, two in 2007, one in 2011, and four individuals in 2017 (Nguyen et al. 2019). To better understand the dietary ecology of the poorly studied black‐eyed bent‐toed gecko, this study aims to provide novel data on food selection of *C. nigriocularis* using the stomach flushing method and compare diet between different sex and age groups. Besides adding to our limited knowledge of this lizard, the results of the study can be used to support future conservation breeding programs of this Critically Endangered taxon, in case anthropogenic threats still pose significant harm to its population.

## 2 Material and Methods

### 2.1 Field Surveys and Sampling

Field surveys were conducted in Ba Den MCHC in September 2022 by H.T Ngo, Q.H Do, H.Q Nguyen, and one local person. Ba Den MCHC, located in Tay Ninh Province, southern Vietnam, was established by Decision No. 1351/QD‐TTg of the Prime Minister, dated 11 July 2016, with an area of 1762.76 ha (The People's Committee of Tay Ninh Province 2021). In terms of climatic conditions, the protected area is located in the tropical climate region of southern Vietnam, with an annual average rainfall of 1.800 mm an annual average temperature of 27°C, and an annual average humidity of 78% (The People's Committee of Tay Ninh Province 2021). A total of three transects were set up based on previous surveys, literature reviews, and interviews with local people and rangers. Coordinates of each sampling locality were taken using a Garmin GPSMap 64SC (WGS 84 datum) and can be shared upon request with the authors. Animals were captured by hand and subsequently released at the collecting site after being photographed, recorded for habitat characteristics, and measured. SVL (snout‐vent length) and MW (mouth width) were taken with a digital caliper to the nearest 0.1 mm, and weight was measured using electronic scales to the nearest 0.1 g. Surveys were undertaken after sunset between 19:00 and 01:00 to guarantee the highest detection probability. During the study, we followed the guidelines approved by the American Society of Ichthyologists and Herpetologists for animal care (Beaupre et al. 2004). The surrounding habitat was granite outcrops with medium hardwoods, shrubs, and arrowroot.

Individuals of *C. nigriocularis* were classified as adult, subadult, or juvenile based on their snout‐vent lengths (SVL) according to the original description and our unpublished data (Nguyen et al. 2006). Sex of adults was determined based on the presence of large swollen hemipenial bulges in males, whereas females' parts were un‐swollen (H. S. Fitch 1987; McDiarmid et al. 2012). A stomach‐flushing technique was used to obtain stomach contents without sacrificing them (Griffiths 1986; Solé et al. 2005; Norval et al. 2012). Forceps, a threaded syringe (60 mL), and infusion tubes of soft material (silicon) were used to collect prey items in the stomach of the species, in particular for small individuals to avoid perforations of the esophagus and stomach. Each individual was stomach‐flushed only once, following the guidelines approved by Beaupre et al. (2004). The water for flushing was taken from bottled drinking water. After stomach‐flushing, animals were monitored for vigor and body conditions and released within 30 min at the place of capture. Prey items were preserved in 70% ethanol (Merck, Germany).

For taxonomic identification, three tail tissue samples were collected and preserved in 70% ethanol (Merck, Germany) for DNA extraction. Total DNA was then extracted using GenJET Genomic DNA Purification kit (Thermo Fisher Scientific, Lithuania) following the manufacturer's instructions. Extraction DNA was amplified by DreamTaq Mastermix (Thermo Fisher Scientific, Lithuania) with 21 μL volume (10 μL of mastermix, 5 μL of water, 2 μL of each primer and 2 μL of total DNA). PCR conditions were 95°C for 5 min to activate the taq; with 35 cycles at 95°C for 30s, 48°C for 45 s, 72°C for 60s; and a final extension at 72°C for 6 min. A fragment of the mitochondrial gene, cytochrome c oxidase subunit I (COI), was amplified using the primer pair VF1d (5′‐TTCTCAACCAACCACAARGAYATYGG‐3′) and VR1d (5′‐TAGACTTCTGGGTGGCCRAARAAYCA‐3′) (Ivanova et al. 2006). PCR products were visualized using electrophoresis through a 2% agarose gel stained with ethidium bromide. Successful amplifications were purified to eliminate PCR components using GenJET PCR Purification kit (Thermo Fisher Scientific, Lithuania). Purified PCR products were sent to FirstBase (Malaysia) for sequencing in both directions. Afterward, sequences were validated with Sequencher v4.10 (Gene Codes, Ann Arbor, MI) with default setting and compared with data available on Genbank using BLAST Tool as implemented in the National Center for Biotechnology Information (NCBI, https://www.ncbi.nlm.nih.gov).

After sequences were aligned by Clustal X v2.1 (Thompson et al. 1997), data were analyzed using maximum likelihood (ML) as implemented in IQ‐TREE v1.6.12 (Nguyen et al. 2015), maximum parsimony (MP) as implemented in PAUP*4.0b10 (Swofford 2001) and Bayesian inference (BI) as implemented in MrBayes v3.2.7 (Ronquist et al. 2012). For the MP analysis, heuristic analysis was conducted with 100 random taxon addition replicates using the tree‐bisection and reconnection (TBR) branch‐swapping algorithm, with no upper limit set for the maximum number of trees saved. Bootstrap support (BP) was calculated using 1000 pseudo‐replicates and 100 random taxon addition replicates. All characters were equally weighted and unordered. For the ML analysis, 10,000 ultrafast bootstrap replications (UFB) were used. The optimal model for nucleotide evolution was determined using jModeltest v1.2.10 (Darriba et al. 2012).

For the BI analysis, we used the optimal model determined by jModeltest with parameters estimated by MrBayes v3.2.7. Two independent analyses with four Markov chains (one cold and three heated) were run simultaneously for 10^7^ generations with a random starting tree and sampled every 1000 generations. Loglikelihood scores of sample points were plotted against generation time to detect stationarity of the Markov chains. Trees generated prior to stationarity were removed from the final analyses using the burn‐in function. The posterior probability values (PP) for all nodes in the final majority rule consensus tree were provided. We regard BP ≥ 70% and UFB ≥ 95 and PP >= 0.95 as strong support and values of < 70%, < 95 and < 0.95, respectively, as weak support (Hillis and Bull 1993; Ronquist et al. 2012; Minh et al. 2013). The optimal model for nucleotide evolution was set to GTR + I + G for BI and analyses. The cut‐off point for the burnin function was set to 25% of the total number of trees generated in Bayesian analysis. Uncorrected pair‐wise divergences were calculated in PAUP*4.0b10.

### 2.2 Stomach Content Analysis

In the laboratory, prey items were identified under a microscope (Olympus SZ 700) following the taxonomic literature of invertebrates (i.e., Naumann et al. 1991; Johnson and Triplehorn 2005; Brusca et al. 2016; Thai 2022). The maximum length (L) and width (W) of each prey item were measured to the nearest 0.1 mm using either a caliper or a calibrated ocular micrometer fitted to a microscope (Hirai and Matsui 2001). The volume (*V*, mm^3^) of the prey item was calculated using the formula for a prolate spheroid (*π* = 3.14; Magnusson et al. 2003):

$$V=\frac{4\pi }{3}*\left(\frac{L}{2}\right)*{\left(\frac{W}{2}\right)}^{2}$$

The index of relative importance (IRI) was used to determine the importance of each food category. This index provides a more informed estimation of prey item consumption than any of the three components alone by using the following formula (Norval et al. 2012):

$$\mathrm{IRI}=\frac{\%F+\%N+\%V}{3}$$

Where *F* is the frequency of prey occurrence in stomachs and *N* is the total number of prey items concerning all prey items.

We used linear regression to examine the relationship between mouth width (MW), snout‐vent length (SVL), body mass (BM), and prey size.

Statistical analyses were performed with the SPSS 20.0 (SPSS Inc., Chicago, Illinois, USA) and with the significance level set to *p* < 0.05 for all analyses. Data are presented as mean ± standard deviation (SD) unless otherwise noted. We used Kendall's tau *b* statistics to examine the relationship between MW, SVL, BM and the prey volume. Wilcoxon's rank sum test (*W*) was used to examine the size of prey items, the number of prey items, and prey volume from frogs of different sexes.

## 3 Results

### 3.1 Species Identification

**FIGURE 1:**
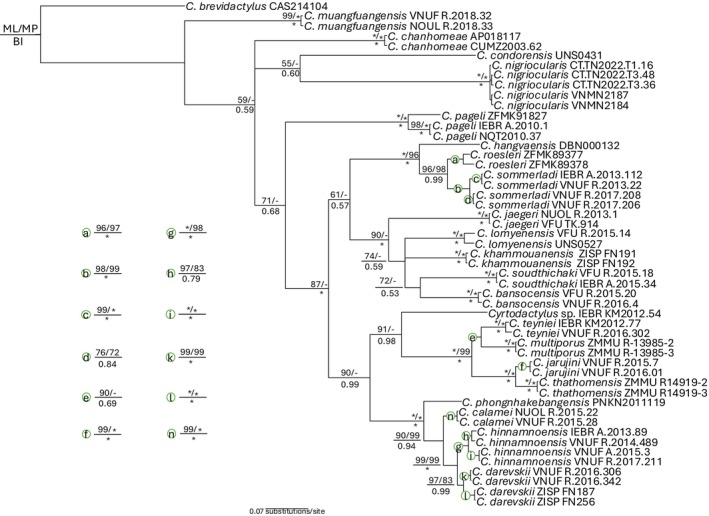
Phylogram based on the Bayesian analysis. Number above and below branches are ML/MP ultrafast bootstrap and bootstrap values and Bayesian posterior probabilities, respectively. Asterisk and hyphen denote 100% in ML and MP or 1 in BI and < 50% values in ML and MP or < 0.5 in BI, respectively.

The matrix of molecular data contained 657 aligned characters, of which 380 were constant, 260 characters were parsimony‐informative. The MP analysis produced a single most parsimonious tree (tree length = 1551; consistency index = 0.31; retention index 0.70). Tree topologies from three analyses, ML, MP and BI, were similar and the *Cyrtodactylus* from Ba Den MCHC, Tay Ninh Province (denoted by CT.TN) were recovered with strong statistical support in all analyses as *C. nigriocularis* (Figure 1). In terms of genetic divergences, all samples of *C. nigriocularis* showed a maximum 0.46% based on a fragment of the mitochondrial COI gene, suggesting they all belong to the same species.

**FIGURE 2:**
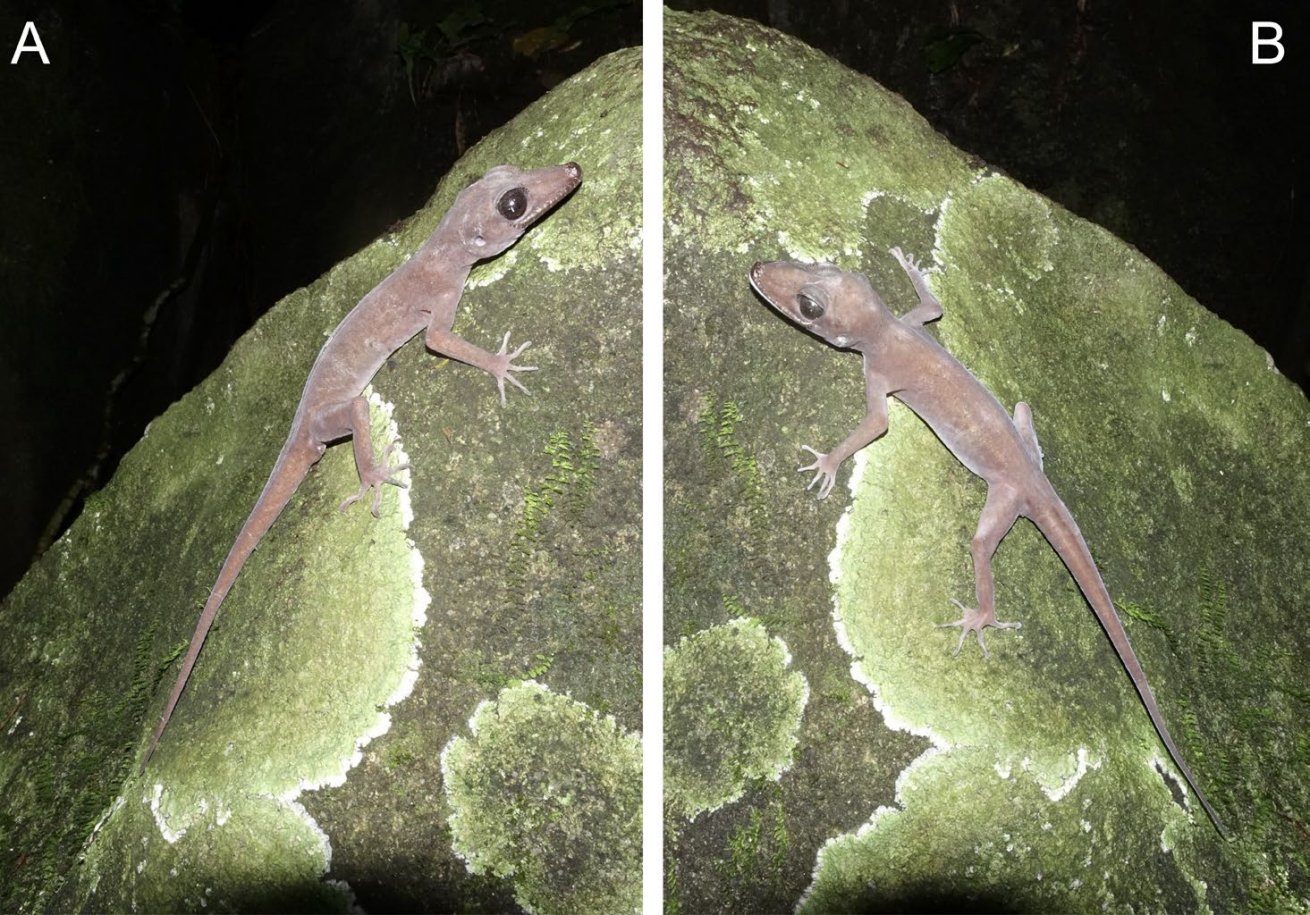
Adult male (A) and adult female (B) of *Cyrtodactylus nigriocularis* in Tay Ninh Province, Vietnam.

**FIGURE 3:**
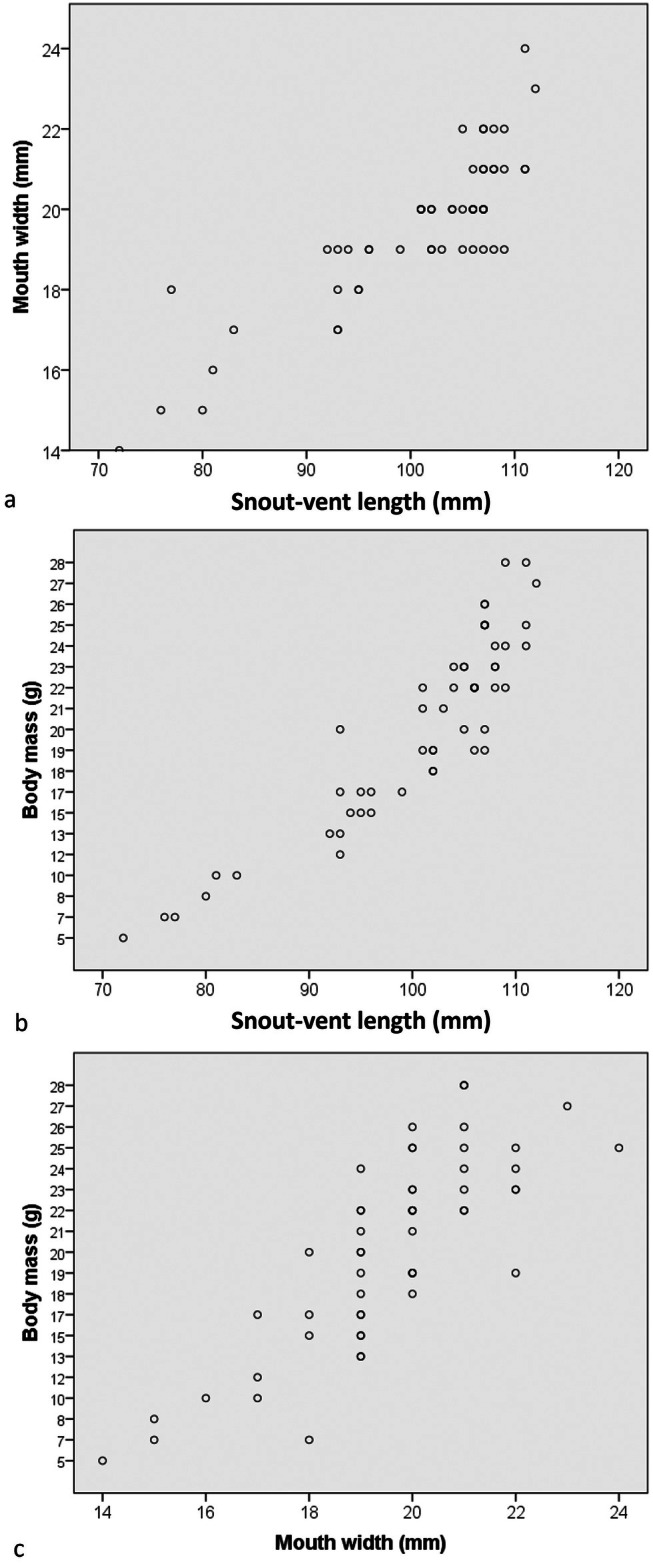
Dispersion diagrams from Pearson's correlations between (a) snout‐vent length and mouth width, (b) snout‐vent length and body mass, and (c) mouth width and body mass of *Cyrtodactylus nigriocularis* in Tay Ninh Province, Vietnam.

Morphological characteristics of the individuals collected in Tay Ninh Province resemble the diagnosis of *C. nigriocularis* (Figure 2; Nguyen et al. 2006) with SVL min–max: 79.77–112.31 mm; mean and SD: 102.26 ± 10.39 mm, *n* = 15, MW 15.13–23.65 mm (20.34 ± 2.35, *n* = 15), and body mass (BM 8.41–26.89 g, 19.93 ± 5.68 g, *n* = 15) in males and SVL 83.20–111.38 mm (102.76 ± 6.23, *n* = 33); MW 16.61–22.10 mm (19.71 ± 1.18, *n* = 33); BM 10.28–28.49 g (20.51 ± 4.60 g, *n* = 33) in females; and SVL: 72.13–76.74 mm; mean and SD: 74.82 ± 2.40 mm, *n* = 3, MW 14.08–17.75 mm (15.61 ± 1.91 mm, *n* = 3), and body mass (BM 5.0–7.20 g, 6.37 ± 1.20 g, *n* = 3) in subadults (Table 1). There were strong positive correlations between the morphological measurements (SVL and MW: *r* = 0.860, *p* < 0.001; SVL and BM: *r* = 0.948; *p* < 0.001; MW and BM: *r* = 0.833, *p* < 0.001; Figure 3a–c).

**TABLE 1:** Measurements (in mm) of *Cyrtodactylus nigriocularis* in Tay Ninh Province, Vietnam.

	SVL	MW	BM
Male	102.26 ± 10.39	20.34 ± 2.35	19.93 ± 5.68
(*n* = 15)	79.77–112.31	15.13–23.65	8.41–26.89
Female	102.76 ± 6.23	19.71 ± 1.18	20.51 ± 4.60
(*n* = 33)	83.20–111.38	16.61–22.10	10.28–28.49
Subadult	74.82 ± 2.40	15.61 ± 1.91	6.37 ± 1.20
(*n* = 3)	72.13–76.74	14.08–17.75	5.0–7.20

### 3.2 Food Items and Prey Dimension

A total of 51 individuals, including 15 males, 33 females, and 3 subadults, of *C. nigriocularis* were captured in Tay Ninh Province for stomach flushing. Overall, 407 prey items (164 items from 15 males, 217 items from 33 females, and 26 items from three subadult) were found. The range number of prey items per individual was 1.0–43.0 (mean and SD: 7.98 ± 8.42 items, *n* = 51). Mean and SD prey item length was 7.53 ± 6.55 mm (min–max: 0.5–61.0 mm, *n* = 407); mean and SD prey item width was 1.44 ± 1.10 mm (min–max: 0.2–8.0 mm, *n* = 407). The average dietary volume per individual was 186.13 ± 343.07 mm^3^ (min–max 0.85–1590.12 mm^3^, *n* = 51).

**FIGURE 4:**
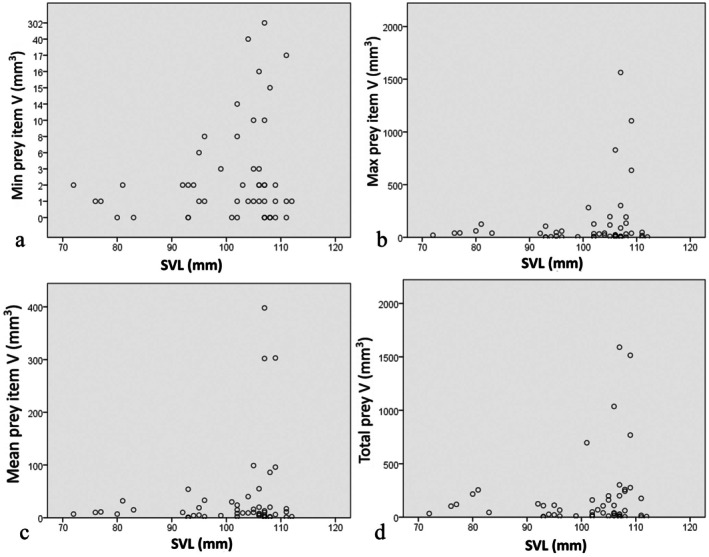
Relationships between the prey volume per stomach (a) snout‐vent length, (b) snout‐vent length, and (c) body mass of *Cyrtodactylus nigriocularis* in Tay Ninh Province, Vietnam.

There was no positive correlation between the bent‐toed gecko SVL and the minimum prey volume (Kendall's tau *b*: tau = −0.023, *p* = 0.818), mean prey item volume (tau = 0.002, *p* = 0.987), maximum prey item volume (tau = −0.015, *p* = 0.879) and the total prey volume (tau = 0.006, *p* = 0.959) (Figure 4a–d).

The median number of prey among individuals with food in their stomachs was 10.93 ± 12.24 for males and 6.58 ± 6.19 for females with no statistically significant intersexual difference (Wilcoxon's rank sum test, *W* = 402.5, *p* = 0.434). Mean prey item length in males was 6.81 ± 6.97 mm (min–max: 1.0–61.0 mm, *n* = 164); ranged from 0.5 to 50.0 mm in females (7.96 ± 6.42 mm, *n* = 217); and 2.0–20.0 mm in subadults (8.44 ± 4.83 mm, *n* = 26). Mean prey item width in males was 1.43 ± 1.07 mm (0.20–7.0 mm, *n* = 164) ranging from 0.5 to 8.0 mm in females (1.48 ± 1.17 mm, *n* = 217); and 0.5–2.0 mm in subadults (1.18 ± 0.49 mm, *n* = 26). The length, width, and volume of prey items were significantly different between females and males (Wilcoxon's rank sum test; length: *W* = 110, *p* < 0.001; width: *W* = 22584.5, *p* < 0.001; volume: *W* = 3490, *p* < 0.001). The average dietary volume per individual in males was 273.36 ± 447.06 mm^3^ (0.85–1590.12 mm^3^, *n* = 15); 155.66 ± 301.42 mm^3^ (1.11–1514.66 mm^3^, *n* = 33) in females (Wilcoxon's rank sum test, sex: *W* = 406.50, *p* = 0.386).

### 3.3 Dietary Diversity

**FIGURE 5:**
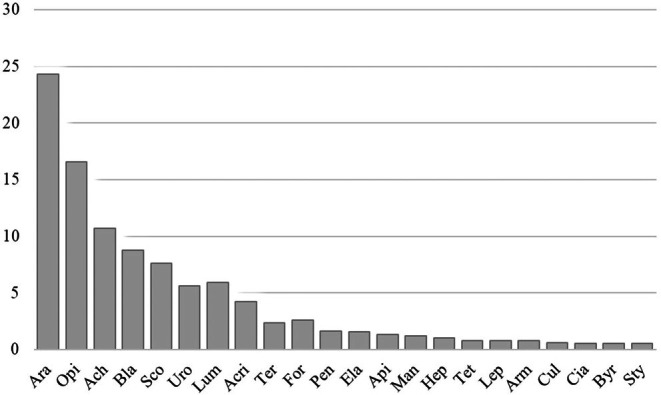
Importance index (IRI) for prey categories of *Cyrtodactylus nigriocularis* in Vietnam. Araneae = Ara, Opiliones = Opi, Blattidae = Bla, Acrididae = Acr, Scolopendridae = Sco, Pentatomidae = Pen, Achatinidae = Ach, Lumbricidae = Lum, Uropigi = Uro, Tetrigidae = Tet, Termitidae = Ter, Formicidae = For, Elateroidea = Ela, Armadillidiidae = Arm, Apidae = Api, Mantidae = Man, Hepialidae = Hep, Lepidoptera other = Lep, Stylommatophora other = Sty, Culicidae = Cul, Cicadellidae = Cia, Byrrhidae = Byr.

We identified 22 prey categories in the stomachs of *C. nigriocularis* . Insects formed the main food component of *C. nigriocularis* , with 14 prey categories (Apidae, Formicidae, Cicadellidae, Blattidae, Byrrhidae, Elateroidea, Culicidae, Hemiptera, Termitidae, Hepialidae, Lepidoptera other, Mantidae, Acrididae, Tetrigidae). Other categories were other invertebrate groups (Araneae, Opiliones, Uropygi, Lumbricidae, Scolopendridae, Armadillidiidae, Achatinidae, Stylommatophora representatives) (Table 2). The highest frequency of occurrence (%F) of prey items identified was Araneae (26.67%), followed by Opiliones (17.33%), Scolopendridae (8.0%), Achatinidae and Blattidae (6.67%), Acrididae (5.33%), while the most proportion (%N) prey group was Araneae (35.87%), followed by Opiliones (23.59%), Blattidae (11.06%), Achatinidae (5.65%), Uropygi and Acrididae (4.67%), Scolopendridae (3.44%). In the comparisons by the IRI (%), Araneae (24.33%), followed by Opiliones (16.59%), Achatinidae (10.67%), Blattidae (8.77%), Scolopendridae (7.59%), and Acrididae (4.20%), were found to be the most important prey groups (Figure 5).

**TABLE 2:** Prey categories consumed by *Cyrtodactylus nigriocularis* in Tay Ninh Province, Vietnam (*n* = 51), (F) total frequency, (%F) relative frequency, (N) total abundance, (%N) relative abundance, (V) total volume (mm^3^), (%V) relative volume; (IRI) importance index.

Prey taxa	F	%F	N	%N	V	%V	IRI
Annelida							
Opisthopora							
Lumbricidae	1	1.33	3	0.74	1493.33	15.73	5.93
Mollusca							
Stylommatophora							
Achatinidae	5	6.67	23	5.65	1869.09	19.69	10.67
Stylommatophora other	1	1.33	1	0.25	1.67	0.02	0.53
Arthropoda							
Arachnida							
Araneae	20	26.67	146	35.87	990.90	10.44	24.33
Opiliones	13	17.33	96	23.59	841.44	8.86	16.59
Uropigi	1	1.33	19	4.67	1035.68	10.91	5.64
Scolopendromorpha							
Scolopendridae	6	8.00	14	3.44	1074.99	11.32	7.59
Isopoda							
Armadillidiidae	1	1.33	2	0.49	39.90	0.42	0.75
Insecta							
Blattodea							
Blattidae	5	6.67	45	11.06	815.22	8.59	8.77
Coleoptera							
Byrrhidae	1	1.33	1	0.25	9.81	0.10	0.56
Elateroidea	1	1.33	1	0.25	302.03	3.18	1.59
Diptera							
Culicidae	1	1.33	1	0.25	14.65	0.15	0.58
Hemiptera							
Cicadellidae	1	1.33	1	0.25	10.47	0.11	0.56
Pentatomidae	2	2.67	2	0.49	159.15	1.68	1.61
Hymenoptera							
Apidae	1	1.33	2	0.49	198.87	2.09	1.31
Formicidae	4	5.33	9	2.21	28.78	0.30	2.62
Isoptera							
Termitidae	3	4.00	10	2.46	63.52	0.67	2.38
Lepidoptera							
Hepialidae	1	1.33	1	0.25	133.97	1.41	1.00
Lepidoptera other	1	1.33	1	0.25	70.52	0.74	0.77
Mantodea							
Mantidae	1	1.33	6	1.47	85.17	0.90	1.23
Orthoptera							
Acrididae	4	5.33	19	4.67	247.76	2.61	4.20
Tetrigidae	1	1.33	4	0.98	5.63	0.06	0.79

## 4 Discussion

The stomach flushing method we used in this study focuses primarily on identifying invertebrates in the dietary items of *C. nigriocularis* . Although this method has some limitations, as it is quite invasive (Luiselli et al. 2011) and cannot account for smaller food items, in the absence of more advanced microscopic and molecular equipment, we opted to employ the practice. Other techniques might include microscopic analysis of pollen grains and other vegetation components in lizards' diet or using more advanced approaches, for example, environmental DNA (eDNA), to determine their food composition (Alemany et al. 2023; Pekár et al. 2023; Pinho et al. 2023; Deso et al. 2024). Using a combination of different methods, especially less invasive ones such as eDNA, can provide more comprehensive and accurate data as well as insights into the trophic niche of the species. Furthermore, because the data collection in this study was limited to a single sampling period (September 2022), seasonal variations in the species diet have not been captured. Future works should clarify the outstanding issues.

Most lizards have often been reported feeding on spiders, beetles, grasshoppers, termites, and ants (Bonfiglio et al. 2006; Rocha and Anjos 2007; Iturriaga and Marrero 2013; Tan et al. 2020). In natural environments, the number and frequency of non‐winged groups (such as spiders, orthopterans, ants, Lepidoptera larvae and termites) account for a comparatively higher volume (Vitt and Zani 1997; Vitt et al. 1997; Zamprogno and Teixeira 1998; Colli et al. 2003; Iturriaga and Marrero 2013). Similarly, in this study, the diet of *C. nigriocularis* was found to mainly consist of arthropods (non‐winged groups), most categorically represented by Araneae, Opiliones, Scolopendridae, and Blattidae. These invertebrates were quite abundant in the study area in Ba Den Mountain MCHC, as we often encountered them during our field surveys. Other studies show that *Cyrtodactylus* can feed on annelid worms, cricket, and insect larvae in both natural and captive conditions (Ellis and Pauwels 2012; Kane et al. 2022). It is thus likely that *Cyrtodactylus* species are opportunistic predators.

Our results are consistent with those reported in previous studies, which show bent‐toed geckos are typical sit‐and‐wait foragers (Huey and Pianka 1981; Ananjeva and Tsellarius 1986). However, there is a significant difference between the diet composition of *C. nigriocularis* and those from other geckos. For example, while the diet of *Coleodactylus natalensis* from the Brazilian Atlantic Forest (*n* = 49) is dominated by arthropods (Isopoda, Araneae, Pseudoscorpiones, Coleoptera, Homoptera, and Collembola) (Lisboa et al. 2012) the most important prey in the diet of *C. nigriocularis* include Araneae, Opiliones, Scolopendridae, Blattidae, and Stylommatophora. Despite the divergence, Araneae makes up the most important prey category for the two species. With regard to *Mediodactylus kotschyi* (Steindachner) in the Mediterranean Insular Ecosystems of the Aegean region, Erstratios and Polyme (1990) found that the diets of one population (*n* = 68) was composed predominantly of Araneida, Thysanura, Coleoptera, Isopoda, Insects larvae, and Pseudoscorpions. Similarly, despite differences in diets between *C. nigriocularis* and *M. kotschyi* , Araneae also constitutes the most important prey category for the two species.

Because the number of individuals in this study is small, it is difficult to support comparisons of the diet by sex and age group. Nonetheless, our preliminary results show that the diets of males and females were slightly different in terms of number of prey items ingested and prey size. In addition, females had a broader trophic spectrum than males (females: 18 prey categories, males: 11 prey categories), possibly because females require more energy for reproduction (Vitt and Caldwell 2009; Iturriaga and Marrero 2013).

Despite the differences in diets between the sexes, all of their main food categories comprise non‐flying invertebrates. Furthermore, Araneae and Opiliones represent the most important prey categories for all groups. The findings need to be confirmed in more in‐depth studies with a larger sample size. Some studies on lizards have shown that morphological differences between males and females might result in differences in diet composition (H. S. Fitch 1978; Schoener et al. 1982; Preest 1994). Furthermore, in this study, we did not find a positive correlation between body size and prey volume, which may indicate that the sample size was not large enough and further research is needed to investigate this issue in more detail. *Cyrtodactylus nigriocularis* inhabits Tay Ninh Province's Ba Den Mountain Cultural and Historical Complex, which receives a lower level of protection compared to a nature reserve.

*C. nigriocularis* is a highly specialized granite cave‐dwelling species known only from just a few small caves in Ma Thien Lanh Valley. It occurs in very low numbers and is extremely susceptible to habitat degradation by tourism activities, road construction, and illegal collecting (Nguyen et al. 2019). Ba Den Mountain has become a popular tourist destination, and a new cable car line was launched a few years ago. Our observation confirms that a new road to the top of the mountain is being constructed, further threatening the species. To our knowledge, there has been no *ex situ* conservation program established for this or any endangered Indochinese bent‐toed geckos anywhere in the world. The data from this study will therefore help inform future captive breeding conservation efforts to safeguard the microendemic and Critically Endangered species from the risk of extinction, especially because feeding a diverse and natural diet can maintain the physical fitness of captive populations (Antwis et al. 2014; Chatpongcharoen et al. 2021; Gao et al. 2023).

## Author Contributions

**Hanh Thi Ngo:** conceptualization (equal), data curation (equal), formal analysis (equal), funding acquisition (equal), investigation (equal), resources (equal). **Minh Le:** conceptualization (equal), data curation (equal), formal analysis (equal), funding acquisition (equal), investigation (equal). **Trang Thu Hoang:** data curation (equal), formal analysis (equal). **Ha Hoang Nguyen:** data curation (equal), formal analysis (equal). **Huy Quoc Nguyen:** investigation (equal). **Quyen Hanh Do:** investigation (equal). **Hanh Minh Vu Nguyen:** data curation (equal), formal analysis (equal). **Truong Quang Nguyen:** methodology (equal), writing – review and editing (equal). **Thomas Ziegler:** writing – review and editing (equal). **Minh Duc Le:** investigation (equal), methodology (equal), writing – review and editing (equal). **Anh Van Pham:** data curation (equal), formal analysis (equal), writing – original draft (equal), writing – review and editing (equal).

## Disclosure

Benefit‐Sharing Statement: This study complies with the principles of the Convention on Biological Diversity and the Nagoya Protocol. A stomach‐flushing technique was used to obtain stomach contents without sacrificing animals. Each individual was stomach‐flushed only once, following the guidelines approved by Beaupre et al. (2004). This research was undertaken in accordance with all national and international regulations on animal welfare. All researchers involved in this study are acknowledged as co‐authors, ensuring fair recognition of their intellectual contributions.

## Conflicts of Interest

The authors declare no conflicts of interest.

## Data Availability

All data and analysis code are available at Zenodo (https://zenodo.org/records/14876916).
